# Supplementary material for: Isotopic evidence for the formation of the Moon in a canonical giant impact
Source: Nat Commun. 2021 Mar 22;12:1817. doi: 10.1038/s41467-021-22155-7 (PMC7985389; doi:10.1038/s41467-021-22155-7)
Supplement: Supplementary file 1 — Supplementary Information [file 41467_2021_22155_MOESM1_ESM.pdf]

Supplement for:

# Isotopic evidence for the formation of the Moon in a canonical giant impact

Sune G. Nielsen[1,2], David V. Bekaert[1] and Maureen Auro[1]

[1]NIRVANA laboratories, Woods Hole Oceanographic Institution, Woods Hole, MA 02543, USA,
[2]Department of Geology and Geophysics, Woods Hole Oceanographic Institution, Woods Hole, MA 02543, USA

## Supplementary Note 1: Correction for GCR production of $^{50}V$ in lunar rocks and chondrites

The V isotope compositions of many lunar rocks are strongly perturbed by galactic cosmic ray (GCR) effects (Fig. 1) that lead to production of $^{50}V$ most likely through interaction with target nuclei of Fe[1]. It is also possible to produce $^{50}V$ enrichments via GCR interactions with isotopes of Ti and Cr, but these have been shown to be very limited, first because Cr cross sections are not very favorable for $^{50}V$ production and secondly because V isotope anomalies in lunar rocks produce poor correlations with Ti/V ratios[1]. Furthermore, V isotope analyses of chondrites reveal significant scatter if corrected using Ti/V ratios abundances, whereas corrections using Fe/V ratios produce entirely invariant V isotope ratios in chondrites[2].

Here we combine all the available lunar V isotope data with their respective cosmic ray exposure (CRE) ages (available in the literature) and Fe/V ratios to produce an empirical calibration line that allows for correction of GCR effects (Fig. 1). The best-fit line through all the data is a York-regression[3] that takes into account all the errors on the CRE ages, Fe/V ratios, and V isotope compositions. The slope of the line is $-2.132\times10^{-6} \pm 0.188\times10^{-6}$ (2SE) and the intercept with the y-axis (e.g., the calculated irradiation-free V isotope composition of the Moon) is $\delta^{51}V_{Moon}$ = -1.037 ± 0.031‰ (2SE). The best-fit curve also includes one lunar soil sample (10084), which, despite a large error on the CRE age, plots on the curve. Although we here include the lunar soil sample in our regression, it is important to note that lunar soil samples in many cases aree not expected to conform to the GCR curve (Fig. 1) because they can be contaminated by chondritic debris from relatively recent impacts. Furthermore, lunar soils are often heterogeneous, which can render different sub-samples with significantly different CRE ages. Hence, lunar soils are generally not ideal samples to construct V isotope GCR corrections from. Here, we do include the one lunar soil sample in our regression because it plots together with 25 other lunar samples. In addition, our regression weights according to sample error bars and, therefore, the lunar soil has a negligible effect on the calculated slope due to the large CRE age uncertainty.

In order to test whether this value is biased by samples with large GCR corrections, we can correct individual samples for irradiation by using the best fit correlation line in Fig. 1. In this approach we only include samples with GCR corrections <0.3‰ (Samples 12004, 12063, 14053, 14321, 15535, 68815, 68115, 74255, 74275, and LAP02205), as smaller GCR corrections are associated with smaller systematic errors. These samples yield an error-weighted average value of $\delta^{51}V_{Moon}$ = -1.033±0.031‰ (n = 10, 2SE), demonstrating that our average GCR-corrected value for all lunar rocks is not biased by samples with large irradiation corrections. Hence, the slope of the GCR correction curve does not have a large impact on the overall value of $\delta^{51}V_{Moon}$. Here, we choose to use the irradiation free intercept in Fig. 1 as the best estimate for the V isotope composition of the Moon ($\delta^{51}V_{Moon}$ = -1.037 ± 0.031; n = 26, 2SE). In section 2 we also discuss two alternative statistical means to calculate the average composition of the Moon in order to emphasize the robustness of the value employed here.

Supplementary table 1 and Supplementary figure 1 show the GCR corrected data and the average V isotope compositions for all lunar rocks measured to date. Samples with larger uncertainties either have large errors for their CRE ages, larger V isotope measurement errors, or both (Supplementary table 1). To investigate the consistency between our V isotope data sets for lunar rocks and that from[1], we calculate the average $\delta^{51}V_{Moon}$ for samples from each of the two studies. In both cases we calculate weighted means of the two data populations whereby samples are assigned a weight of one divided by the total propagated error squared. The two error-weighted average values are remarkably similar at $\delta^{51}V$ = -1.012 ± 0.067 (n = 16, 2SE) and $\delta^{51}V$ = -1.041 ±

0.030‰ (n = 10, 2SE), which implies excellent agreement between both studies. This conclusion is further supported by Monte Carlo simulations, whereby raw data (actual values and their associated uncertainties) are used to artificially generate 1 million datasets for each study. At each iteration step, the difference of mean $\delta^{51}V$ between the two studies is computed as $\Delta^{51}V_{HPK\text{-}TS} = \delta^{51}V_{HPK} - \delta^{51}V_{TS.}$, where "HPK" and "TS" refer to Hopkins et al.[ref 1] and this study, respectively. The outcomes of this simulation are reported in Supplementary figure 2, supporting the excellent agreement between the two datasets, with a mean $\Delta^{51}V_{HPK\text{-}TS}$ value very close to zero (i.e., no statistical difference).

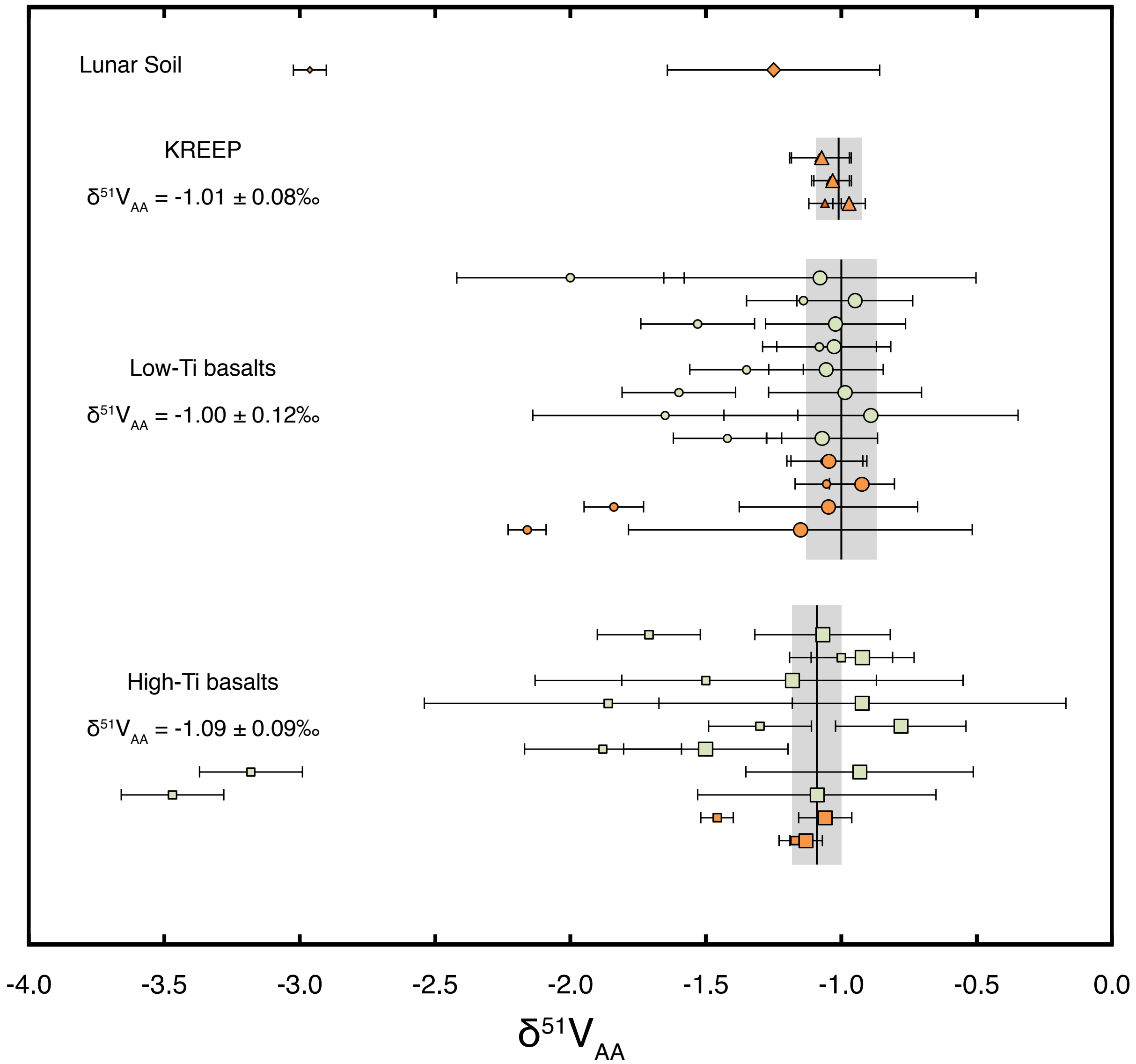


**Supplementary figure 1:** Plot of uncorrected (small symbols) and irradiation corrected (large symbols) lunar vanadium isotope data. Data are grouped into Lunar soils (diamonds), KREEP (triangles), Low-Ti basalts (circles), and High-Ti basalts (squares). Green samples are literature data[1], orange data are form this study. Grey bars denote weighted averages and 2SE uncertainties of each sample group. Data are listed in Supplementary Table 1.

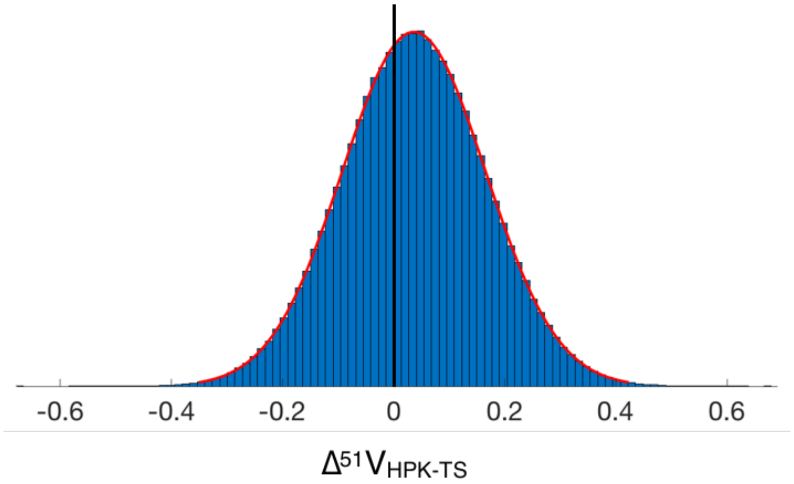


**Supplementary figure 2:** Difference in the mean $\delta^{51}V$ of the Moon computed from datasets of ref. 1 and this study ($\Delta^{51}V_{HPK-TS}$), as derived from 1 million runs of Monte Carlo simulation.

## Supplementary Note 2: The vanadium isotopic difference between the BSE and the Moon

The difference between the mean $\delta^{51}V$ value of the Moon and BSE is small, being ~0.2 ‰, but resolvable at our present level of precision. To demonstrate and quantify this difference, we present a series of statistical considerations. Supplementary figure 3 shows the densities we obtained through Kernel density estimation, as well as the histograms and corresponding Gaussian fits for the Moon and BSE vanadium isotope data. It is notable that the density of Moon $\delta^{51}V$ statistics is shifted towards the left-hand side with respect to the density of Earth statistics. This suggests that Moon and Earth $\delta^{51}V$ statistics do not have the same means and that the mean for the Moon is lower.

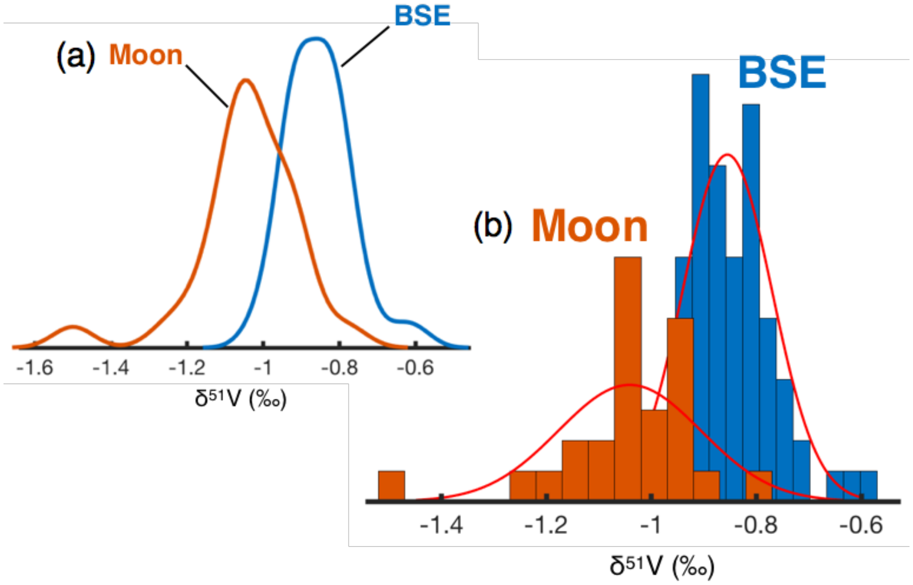


**Supplementary figure 3:** Comparison between the V isotopic datasets for the Moon and BSE, with (a) Kernel estimate densities and (b) frequency distributions showed as histograms with their associated Gaussian best fits.

Here, we carried out Monte Carlo simulations using the raw data for the BSE and Moon data sets and their associated individual uncertainties to artificially generate 1 million datasets, and hence compute 1 million $\delta^{51}V$ mean values for the Moon and BSE. At each iteration step, the difference between the mean $\delta^{51}V$ of the BSE and the Moon was computed as $\Delta^{51}V_{BSE\text{-}Moon} = \delta^{51}V_{BSE} - \delta^{51}V_{Moon}$. The distribution of $\Delta^{51}V_{BSE\text{-}Moon}$ values is shown in Supplementary figure 4, demonstrating that the probability for the silicate Earth to have a higher mean $\delta^{51}V$ than the Moon (i.e., $\Delta^{51}V_{BSE\text{-}Moon} > 0$) is extremely high (99.67%). These simulations imply that a shift of 0.186 ± 0.068 (1SD) exists between the V isotope compositions of the Moon and BSE (Supplementary figure 7). The corresponding mean $\delta^{51}V$ of the BSE, Chondrites and Moon are $\delta^{51}V_{BSE} = -0.855 \pm 0.012$ (1SD), $\delta^{51}V_{ch.} = -1.094 \pm 0.038$ (1SD), and $\delta^{51}V_{Moon} = -1.041 \pm 0.067$ (1SD), respectively.

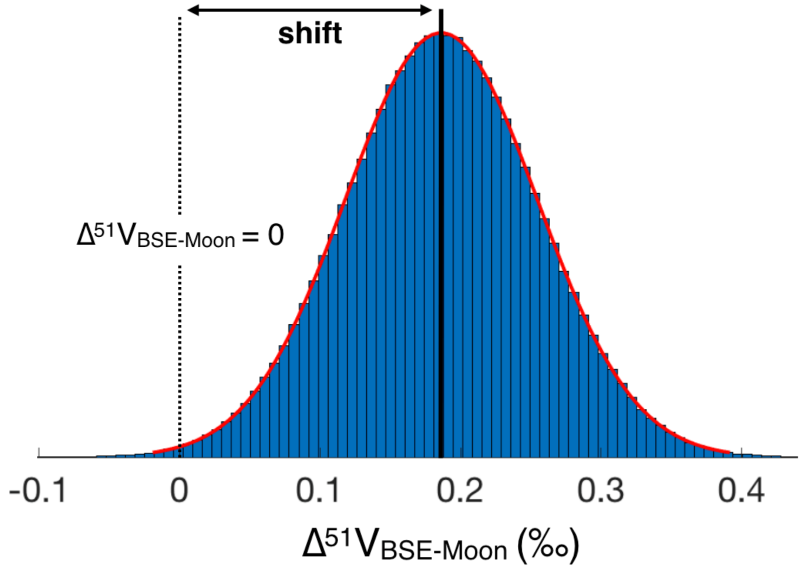


**Supplementary figure 4:** Frequency distribution of the V isotope difference between the BSE and the Moon ($\Delta^{51}V_{BSE\text{-}Moon}$) as generated by Monte Carlo simulations where the raw data and their associated uncertainties are used to artificially generate 1 million mean $\delta^{51}V_{BSE}$ and $\delta^{51}V_{Moon}$ values.

When calculating the mean values and error weighted 2SE for each of BSE, chondrites, and the Moon, it is necessary to make the assumption that all three populations are normally distributed. One first-order observation that supports this inference is that all individual samples in the chondrite[2] and Moon datasets (once corrected for GCR effects) exhibit V isotope compositions that are all within error of each other. The same is true for 92% of the samples representing BSE, which is expected within the 95% confidence interval for any given data population. However, we can further perform a series of tests to investigate whether or not the corresponding datasets are normally distributed and, hence, allow us to demonstrate the statistical validity of the calculated averages and associated errors for each data population. In this approach, the main limiting factor corresponds to the small amount of available data for each end-member. However, note that the frequency distributions of $\delta^{51}V_{BSE}$ and $\delta^{51}V_{Moon}$ broadly follow Gaussian distributions (Supplementary figure 3). Furthermore, quantile-quantile (Q-Q) plots of our datasets are broadly consistent with Gaussian distributions for all three end-members (Supplementary figure 5). These plots correspond to a graphical method for comparing two probability distributions - here the quantiles of the sample datasets versus theoretical quantile values generated from normal

distributions - by plotting their quantiles against each other. However, these graphical considerations do not allow quantitatively validating/refuting the hypothesis of our datasets being normally distributed, and further statistical tests are therefore required.

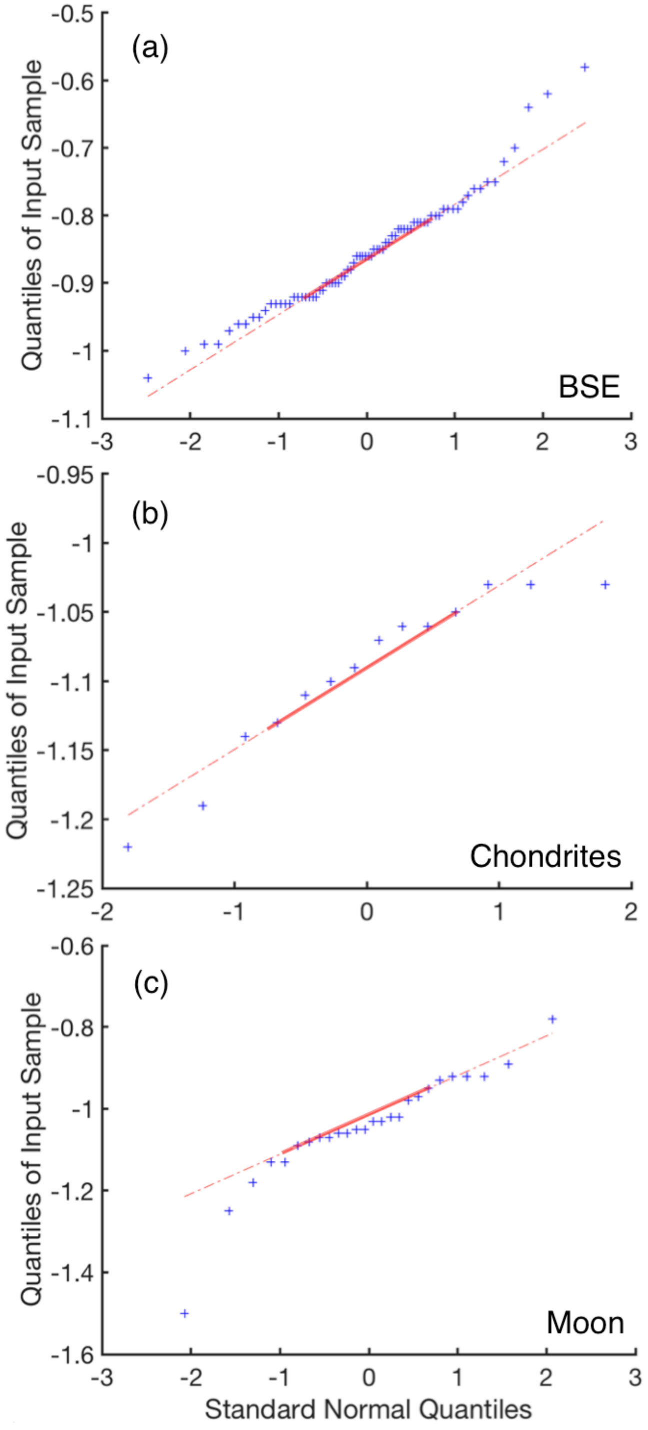


**Supplementary figure 5:** Q-Q plots comparing the probability distributions of the BSE, Chondrites and Moon V isotope raw data (Supplementary Table 1 and ref [2]) with normal distributions.

First, we postulate the null hypothesis that our three sets of data come from normal distributions with mean compositions of $\delta^{51}V_{BSE}$ = -0.86, $\delta^{51}V_{ch.}$ = -1.09, $\delta^{51}V_{Moon}$ = -1.04, and unknown variances. Student's t-tests carried out for each dataset do not reject the null hypothesis at the 5% significance level ($\alpha$ = 0.05). In order to compensate for the small size of our datasets and further investigate the reliability of these results, we used Monte Carlo simulations to artificially generate 1 million datasets for the BSE, chondrites and Moon from the raw data and associated uncertainties. We found that Student's t-test rates of success (i.e., no rejection of the null hypothesis at the 5% significance level) of 98.50%, 96.75% and 96.97% for the BSE, chondrites and Moon, respectively, indicating that Student's t-tests consistently fail to reject the null hypothesis.

We further tested the null hypothesis by carrying Lilliefors tests, which correspond to a test for goodness of fit to a normal distribution based on the Kolmogorov–Smirnov test. Unlike Student's t-tests, Lilliefors tests return p-values, which represent the probability of observing a test statistic as extreme as, or more extreme than, the observed value under the null hypothesis. In other words, small values of p cast doubt on the validity of the null hypothesis. If the p-value is less than or equal to $\alpha$ (i.e., $p \leq 0.05$), then the Lilliefors test rejects the null hypothesis. If the p-value is greater than $\alpha$ (i.e., $p > 0.05$), then the Lilliefors test fails to reject the null hypothesis. Here, we again used Monte Carlo simulations to artificially generate 1 million datasets for the BSE, chondrites and Moon from the raw data and associated uncertainties. We found that Lilliefors tests give mean p-values of 0.106, 0.209 and 0.131, with rates of success (i.e., no rejection of the null hypothesis) of 52.33%, 82.01% and 60.44% for the BSE, chondrites and Moon, respectively. Although these results cannot demonstrate that our datasets are normally distributed, they support the absence of significant evidence for them not to be normally distributed. In the absence of statistical arguments to reject the null hypothesis, and in light of all graphical and numerical considerations presented in this section, it appears justified to define the BSE, Moon and chondrite end-members by their "real" values (i.e., using error-weighted mean and 95% c.i.).

Error-weighted $\delta^{51}V$ means are computed using IsoplotR. These give $\delta^{51}V_{BSE}$ = -0.861 ± 0.005 (n = 76, 2SE, MSWD=10.165), $\delta^{51}V_{ch.}$ = -1.089 ± 0.029 (n = 14, 2SE, MSWD=0.690), and $\delta^{51}V_{Moon}$ = -1.036 ± 0.028 (n = 26, 2SE, MSWD=1.539). MSWD values close to 1 for Chondrites and the Moon indicate that error-weighted means and their associated errors are representative of the raw data dispersion. In addition, this $\delta^{51}V_{Moon}$ value is in excellent agreement with the irradiation-free composition of the Moon as derived from the y-intercept of the best fit line to lunar V isotope data plotted against their respective CRE ages ($\delta^{51}V$ = -1.037 ± 0.031‰; n = 26, 2SE, Supplementary figure 1). However, the high MSWD value for the BSE (>>1) indicates that $\delta^{51}V_{BSE}$ values are overdispersed with respect to the stated analytical uncertainties, implying that the error-weighted mean and 2SE cannot be used most likely due to individual uncertainties being underestimated. This inference is also supported by the fact that some studies have reported individual sample uncertainties far lower than the long-term external reproducibility of the V isotope measurement technique[4,5]. We therefore compute the unweighted mean of the $\delta^{51}V_{BSE}$, which gives $\delta^{51}V_{BSE}$ = -0.856 ± 0.020 (2SE, n=76). Computing the mean and 2SE of the $\delta^{51}V_{BSE}$ from the mean $\delta^{51}V$ and 2SE of peridotites, MORBs, komatiites and OIBs produces a MSWD of 0.77, which is suggestive of uncertainties being representative of the overall data dispersion. The mean and 2SE of the $\delta^{51}V_{BSE}$ (n=76) is, hence, used for mixing calculations (see section 3).

We ran Monte Carlo simulations (1 million runs) to investigate the range of possible $\Delta^{51}V_{BSE\text{-}Moon}$ using $\delta^{51}V_{BSE}$ = -0.856 ± 0.020 and $\delta^{51}V_{Moon}$ = -1.037 ± 0.031. These give mean $\Delta^{51}V_{BSE\text{-}Moon}$ of 0.180 ± 0.035 (2SE; Supplementary figure 6), in good agreement with our estimate from Monte

Carlo simulations using raw data to artificially generate $\delta^{51}V$ datasets for the Earth and Moon (0.186 ± 0.068, 1SD; Supplementary figure 4).

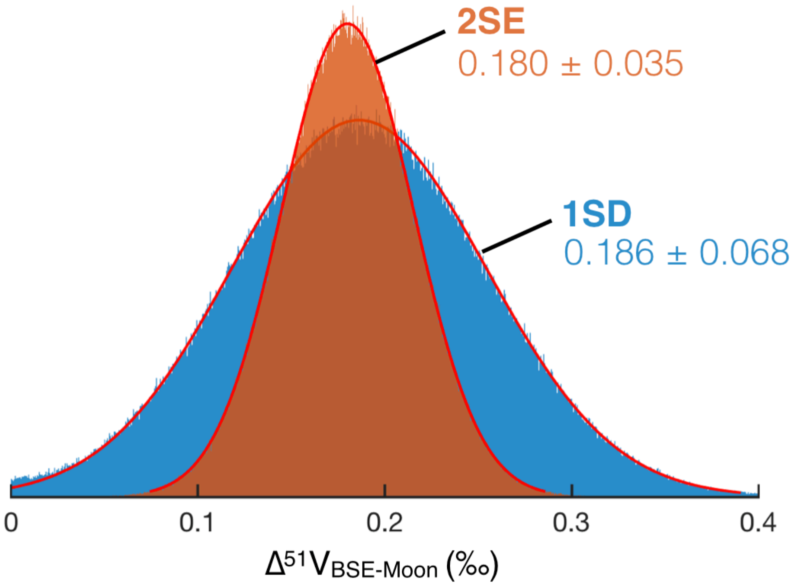


**Supplementary figure 6:** Frequency distribution of the V isotope difference between the BSE and the Moon ($\Delta^{51}V_{BSE\text{-}Moon}$) as generated by Monte Carlo simulations. In the first case (blue plot), the raw data and their associated uncertainties are used to artificially generate 1 million mean $\delta^{51}V_{BSE}$ and $\delta^{51}V_{Moon}$ values and compute $\Delta^{51}V_{BSE\text{-}Moon}$ (cf. Supplementary figure 4). In the second case (orange plot), our estimated "real values" for the $\delta^{51}V$ of the Moon and BSE and associated 2SE are used as input parameters for Monte Carlo simulations.

**In conclusion,** we have adopted two complementary approaches to compute the V isotope difference between the Moon and BSE. The first one (**A1**) relies on Monte Carlo simulations using the raw data and their associated uncertainties to artificially generate 1 million datasets and compute mean V isotope compositions for the Moon, BSE and chondrites. This approach yields $\Delta^{51}V_{BSE\text{-}Moon}$ = 0.186 ± 0.068 (1SD), $\delta^{51}V_{BSE}$ = -0.855 ± 0.012 (1SD), $\delta^{51}V_{ch.}$ = -1.094 ± 0.038 (1SD), and $\delta^{51}V_{Moon}$ = -1.041 ± 0.067 (1SD). The second approach (**A2**) uses the means and 2SE of the three end-members. This approach yields $\Delta^{51}V_{BSE\text{-}Moon}$ = 0.180 ± 0.035 (2SE), $\delta^{51}V_{BSE}$ = -0.856 ± 0.020 (2SE), $\delta^{51}V_{ch.}$ = -1.089 ± 0.029 (2SE), and $\delta^{51}V_{Moon}$ = -1.036 ± 0.028 (2SE). The results of these two approaches are in very good agreement with each other, and are summarized in Supplementary figure 7.

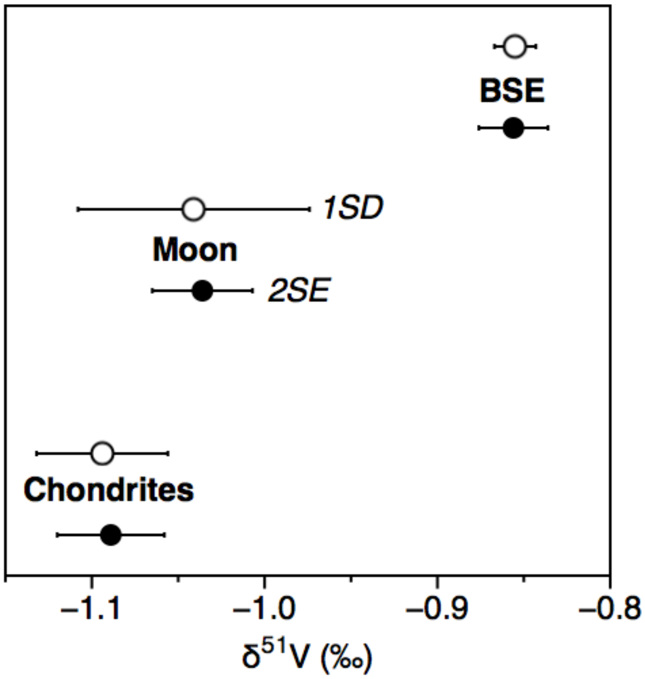


**Supplementary figure 7:** Comparison between the mean V isotopic compositions of the Moon, chondrites and BSE from (i) the raw data-driven Monte Carlo approach (open symbols, errors bars at 1SD), and (ii) computation of the "real" values of these end-members (closed symbols).

## Supplementary Note 3: Vanadium isotope constraints the Moon-forming event

Mixing calculations presented in this paper rely on the fundemental principle of mass conservation during two-component mixing, for a system with pre-impact (proto-Earth, Theia) and post-impact (Earth, Moon, escaping mass (EM)) components (see ref [6] for description of a similar approach). We consider $\delta^{51}V_{P\text{-}E}$, $\delta^{51}V_{Theia}$, $\delta^{51}V_{BSE}$, and $\delta^{51}V_{Moon}$ the $\delta^{51}V$ values of the proto-Earth, Theia, BSE and Moon, respectively. The $\delta^{51}V_{BSE} = -0.856 \pm 0.020$ (2SE), $\delta^{51}V_{ch.} = -1.089 \pm 0.029$ (2SE), and $\delta^{51}V_{Moon} = -1.037 \pm 0.031$ (2SE) are taken from the respective best estimates for each reservoir, which yields a best estimate for the V isotope composition difference between BSE and the Moon of $\Delta^{51}V_{BSE\text{-}Moon} = 0.180 \pm 0.035$ (2SE). The V isotope composition of Theia is assumed to be indistinguishable from chondrites ($\delta^{51}V_{Theia} = \delta^{51}V_{ch.}$), which is conventionally assumed in most Giant Impact simulations and also reasonable given that all chondrite groups have identical V isotope compositions[2]. Given that V is only mildly siderophile and, therefore, core formation does not tend to produce a silicate portion of a planet with concentrations significantly different to bulk chondrites, we here assume that the proto-Earth, Theia and the Moon all have similar V concentrations. Most chondrites have V concentrations that are within error of BSE (82±12 µg/g [2]). So far we only have V data for two enstatite chondrites and these suggest the potential for the chondritic impactor to have had lower V concentrations than considered here (~50 µg/g [2]). However, a lower V concentration for the impactor would only change our model in a minor way, and would invariably lead to slightly higher mass fractions of Theia ($\varphi_M$) being required in the Moon to account for its present-day V isotope composition. Thus, a lower V concentration for

Theia would further strengthen our conclusion that the observed $\Delta^{51}V_{BSE\text{-}Moon}$ is only compatible with the canonical giant impact simulations. The mass fractions of the present-day Moon and Earth that originate from Theia are denoted $\varphi_M$ and $\varphi_E$, respectively, with:

$$\delta^{51}V_{BSE} = \varphi_E * \delta^{51}V_{Theia} + (1\text{-} \varphi_E) * \delta^{51}V_{P\text{-}E} \quad \text{(Eq. 1)}$$

$$\delta^{51}V_{Moon} = \varphi_M * \delta^{51}V_{Theia} + (1\text{-} \varphi_M) * \delta^{51}V_{P\text{-}E} \quad \text{(Eq. 2)}$$

These two equations contain 3 unknowns, which are $\varphi_E$, $\varphi_M$ and $\delta^{51}V_{P\text{-}E}$. Mass conservation implies:

$$M_{Theia} = \varphi_E * M_{Earth} + \varphi_M * M_{Moon} + EM \quad \text{(Eq. 3)}$$

where $M_{Theia}$, $M_{Earth}$ and $M_{Moon}$ are the total masses of Theia, Earth and Moon, respectively. Whereas the two latter are known, $M_{Theia}$ is taken as a free parameter that will be varied from 0.8 * $M_{Mars}$ to 0.5 * $M_{Earth}$, where $M_{Mars}$ corresponds to the total mass of Mars. In this approach, the *EM* (ejected mass) term can be neglected, as only the fraction of Theia's material ultimately entering either the Moon or Earth is considered in the mass balance. In other words, two scenarios where $M_{Theia}$ = 1.2 * $M_{Mars}$ and $EM$ = 0.2 * $M_{Mars}$, or $M_{Theia}$ = 1.0 * $M_{Mars}$ and $EM$ = 0 * $M_{Mars}$ will give the exact same outcomes. The system of Eq. 1-3 hence contains 3 unknowns, and can therefore be fully resolved to investigate the ranges of admissible $\varphi_E$, $\varphi_M$ and $\delta^{51}V_{P\text{-}E}$ values as constrained by V isotope systematics. We also note $\varphi_{M\text{-}T}$ the fraction of Theia that ends up being incorporated in the Moon:

$$\varphi_{T\text{-}M} = \varphi_M * M_{Moon} / M_{Theia} \quad \text{(Eq. 4)}$$

A summary of the main outcomes of these mixing calculations is reported in Table 1, whereas Fig. 3 of the main manuscript presents illustrations of the mixing calculations where $M_{Theia}$ represents 0.8*$M_{Mars}$, 1.2*$M_{Mars}$, and $M_{Theia}$ = 0.45*$M_{Earth}$. As illustrated in Supplementary figure 8, using $\delta^{51}V_{BSE}$, $\delta^{51}V_{Theia}$, $\delta^{51}V_{Moon}$ and $\Delta^{51}V_{BSE\text{-}Moon}$ values as derived from our first approach A1 would essentially give the same outcomes (at the 1SD level) as what is presented in the manuscript. This supports our conclusion that V isotope systematics for lunar samples requires the Moon to be predominantly derived from the impactor, as predicted by the canonical giant impact scenario.

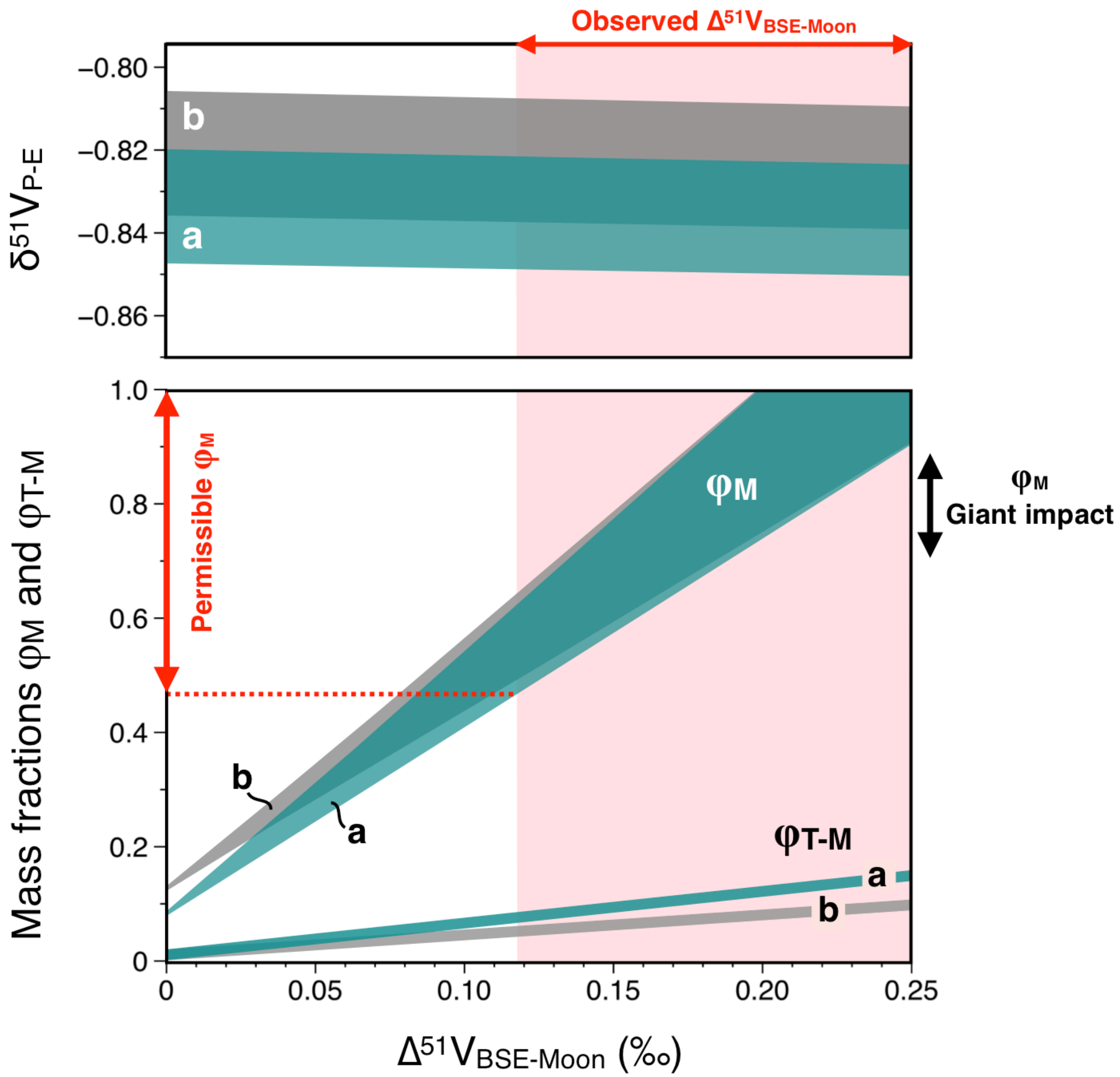


**Supplementary figure 8:** Results of mass balance calculations for giant impacts where Theia's mass ($M_{Theia}$) represents 0.8 (a) or 1.2 (b) times that of Mars ($M_{Mars}$), using end-member values from approach A1 (instead of approach A2, as presented in Figure 3 of the main manuscript). Given the observed $\Delta^{51}V_{BSE\text{-}Moon} = 0.186 \pm 0.068$ (1SD), it can be seen that the minimum fraction of Theia in the Moon is ~50%, in very good agreement with constraints from Figure 3 of the main manuscript based on end-member compositions from approach A2. $\varphi_M$ : mass fraction of the present-day Moon that originates from Theia. $\varphi_{T\text{-}M}$ : mass fraction of Theia that has been incorporated in the Moon ($= \varphi_M * M_{Moon} / M_{Theia}$). $\delta^{51}V_{P\text{-}E}$ : V isotopic composition of the proto-Earth.

**Supplementary Table 1:** Vanadium isotope compositions and GCR corrections for lunar samples.

| Sample | Type | V (µg/g) | Fe (µg/g) | CRE age (Myr)[§] | error (2sd) | $\delta^{51}V_{meas}$ | error (2sd) | n | $\delta^{51}V_{corr}$ | error[*] (2sd) |
|---|---|---|---|---|---|---|---|---|---|---|
| Hopkins et al 2019 | | | | | | | | | | |
| 10017 | High-Ti basalt | 68 | 157635 | 479 | 60 | -3.47 | 0.19 | 1 | -1.09 | 0.35 |
| 10017 | High-Ti basalt | 70 | 154955 | 479 | 60 | -3.18 | 0.19 | 1 | -0.93 | 0.34 |
| 10020 | High-Ti basalt | 104 | 146310 | 127 | 25 | -1.88 | 0.29 | 2 | -1.50 | 0.30 |
| 10044 | High-Ti basalt | 42 | 147476 | 70 | 17 | -1.30 | 0.19 | 3 | -0.78 | 0.23 |
| 70255 | High-Ti basalt | 92 | 144242 | 280 | 85 | -1.86 | 0.68 | 3 | -0.92 | 0.74 |
| 71135 | High-Ti basalt | 101 | 144756 | 103 | 3 | -1.50 | 0.63 | 3 | -1.18 | 0.63 |
| 74275 | High-Ti basalt | 118 | 141629 | 32 | 1 | -1.00 | 0.19 | 3 | -0.92 | 0.19 |
| 75035 | High-Ti basalt | 37 | 143636 | 77.4 | 19.3 | -1.71 | 0.19 | 2 | -1.07 | 0.25 |
| 12018 | Low-Ti basalt | 182 | 160419 | 195 | 16 | -1.42 | 0.20 | 3 | -1.05 | 0.20 |
| 12054 | Low-Ti basalt | 118 | 161584 | 260 | 70 | -1.65 | 0.49 | 3 | -0.89 | 0.53 |
| 12054 | Low-Ti basalt | 143 | 158737 | 260 | 70 | -1.60 | 0.21 | 4 | -0.98 | 0.27 |
| 12063 | Low-Ti basalt | 115 | 166756 | 95 | 5 | -1.35 | 0.21 | 3 | -1.06 | 0.21 |
| 14053 | Low-Ti basalt | 108 | 133525 | 21.2 | 5 | -1.08 | 0.21 | 3 | -1.02 | 0.21 |
| 15016 | Low-Ti basalt | 234 | 173167 | 322 | 83 | -1.53 | 0.21 | 2 | -1.02 | 0.25 |
| 15535 | Low-Ti basalt | 217 | 178968 | 110 | 20 | -1.14 | 0.21 | 2 | -0.95 | 0.21 |
| 15556 | Low-Ti basalt | 211 | 166707 | 546 | 214 | -2.00 | 0.42 | 2 | -1.08 | 0.55 |
| | | | | | weighted average[#] | | | | -1.012 | |
| | | | | | weighted 2SE | | | | 0.067 | |
| This study | | | | | | | | | | |
| 10084 | Soil | 53 | 81177 | 520 | 120 | -2.96 | 0.06 | 8 | -1.25 | 0.40 |
| 12004 | Low-Ti basalt | 117 | 148628 | 49 | 10 | -1.05 | 0.12 | 7 | -0.92 | 0.12 |
| 15495 | Low-Ti basalt | 101 | 151612 | 320 | 200 | -2.16 | 0.07 | 3 | -1.13 | 0.65 |
| 15556 | Low-Ti basalt | 224 | 155451 | 546 | 214 | -1.84 | 0.11 | 4 | -1.03 | 0.33 |
| LAP02205 | Low-Ti basalt | 99 | 171363 | 4 | 1 | -1.06 | 0.14 | 3 | -1.05 | 0.14 |
| 70215 | High-Ti basalt | 94 | 139463 | 126 | 3 | -1.46 | 0.06 | 4 | -1.06 | 0.10 |
| 74255 | High-Ti basalt | 102 | 102272 | 17 | 2 | -1.17 | 0.06 | 4 | -1.13 | 0.06 |
| 68815 | KREEP-rich | 21 | 40272 | 2.04 | 0.2 | -1.04 | 0.07 | 4 | -1.03 | 0.07 |
| 68115 | KREEP-rich | 21 | 32458 | 2.08 | 0.08 | -1.08 | 0.11 | 4 | -1.07 | 0.11 |
| 14321 | KREEP-rich | 56 | 100850 | 23.8 | 2 | -1.06 | 0.06 | 4 | -0.97 | 0.06 |
| | | | | | weighted average[#] | | | | -1.041 | |
| | | | | | weighted 2SE | | | | 0.030 | |

[*] - total propagated error combines uncertainties on V isotope measurement, CRE age, Fe and V concentrations.
[#] - Averages and associated 2 sigma errors weighted by total propagated error on each measurement
[§] - CRE ages are based on noble gas isotope measurements in each sample[1,7-15], except for 15495 that is based on range of CRE ages for samples at the edge of the Dune Crater where this sample was collected[8]

**References**

1 Hopkins, S. S. *et al.* The vanadium isotopic composition of lunar basalts. *Earth Planet. Sci. Lett.* **511**, 12-24 (2019).

2 Nielsen, S. G., Bekaert, D. V., Magna, T., Mezger, K. & Auro, M. The Vanadium Isotope Composition of Mars: Implications for Planetary Differentiation in the Early Solar System *Geochemical Perspectives Letters* **15**, 35-39 (2020).

3 York, D., Evensen, N. M., Martinez, M. L. & Delgado, J. D. Unified equations for the slope, intercept, and standard errors of the best straight line. *Am J Phys* **72**, 367-375, doi:10.1119/1.1632486 (2004).

4 Qi, Y. H. *et al.* The vanadium isotopic composition of the BSE: constraints from peridotites and komatiites. *Geochim. Cosmochim. Acta.* **259**, 288-301, doi:10.1016/j.gca.2019.06.008 (2019).

5 Wu, F. *et al.* Vanadium isotope compositions of mid-ocean ridge lavas and altered oceanic crust. *Earth Planet. Sci. Lett.* **493**, 128-139, doi:https://doi.org/10.1016/j.epsl.2018.04.009 (2018).

6 Akram, W. & Schönbächler, M. Zirconium isotope constraints on the composition of Theia and current Moon-forming theories. *Earth Planet. Sci. Lett.* **449**, 302-310, doi:https://doi.org/10.1016/j.epsl.2016.05.022 (2016).

7 Shuster, D. L. & Cassata, W. S. Paleotemperatures at the lunar surfaces from open system behavior of cosmogenic 38Ar and radiogenic 40Ar. *Geochim. Cosmochim. Acta.* **155**, 154-171, doi:https://doi.org/10.1016/j.gca.2015.01.037 (2015).

8 Husain, L. 40Ar-39Ar chronology and cosmic ray exposure ages of the Apollo 15 samples. *Journal of Geophysical Research (1896-1977)* **79**, 2588-2606, doi:doi:10.1029/JB079i017p02588 (1974).

9 Jordan, J. L., Heymann, D. & Lakatos, S. Inert gas patterns in the regolith at the Apollo 15 landing site. *Geochim. Cosmochim. Acta.* **38**, 65-78, doi:https://doi.org/10.1016/0016-7037(74)90195-1 (1974).

10 Eberhardt, P. *et al.* Trapped solar wind noble gases, exposure age and K/Ar-age in Apollo 11 lunar fine material, in *Proceedings of the Apollo 11 Lunar Science Conference.* 1037-1070.

11 Drozd, R. J., Hohenberg, C. M., Morgan, C. J. & Ralston, C. E. Cosmic-ray exposure history at the Apollo 16 and other lunar sites: lunar surface dynamics. *Geochim. Cosmochim. Acta.* **38**, 1625-1642, doi:https://doi.org/10.1016/0016-7037(74)90178-1 (1974).

12 Lugmair, G. W. & Marti, K. Exposure Ages and Neutron Capture Record in Lunar Samples from Fra Mauro, in *Proceedings of the third Lunar Science Conference.* 1891-1897.

13 Eugster, O. *et al.* The cosmic-ray exposure history of Shorty Crater samples; The age of Shorty Crater, in *Proceedings of the eigth Lunar Science Conference.* 3059-3082.

14 Drozd, R. J., Hohenberg, C. M., Morgan, C. J., Podosek, F. A. & Wroge, M. L. Cosmic Ray Exposure History at Taurus-Littrow, in *Proceedings of the 8th Lunar Science Conference.* 3027-3043.

15 Hintenberger, H., Weber, H. W. & Takaoka, N. Concentrations and isotopic abundances of the rare gases in lunar matter, in *Proceedings of the second Lunar Science Conference.* 1607-1625.
